# Self-medication practices and rational drug use habits among university students: a cross-sectional study from Kahramanmaraş, Turkey

**DOI:** 10.7717/peerj.3990

**Published:** 2017-11-01

**Authors:** Ramazan Azim Okyay, Ayşegül Erdoğan

**Affiliations:** Faculty of Medicine, Department of Public Health, Kahramanmaraş Sütçü İmam University, Kahramanmaraş, Turkey

**Keywords:** Self-medication, Rational use of drugs, University students, Turkey

## Abstract

**Background:**

Self-medication refers to the use of medicines to treat self-diagnosed diseases without consulting any healthcare professionals. Irrational drug use and self-medication have serious negative consequences both on health and economy. Therefore, the aim of this study is to assess the habits related to rational use of drugs (RUD) and to estimate the prevalence of self-medication practices among university students.

**Methods:**

This cross-sectional study was conducted on university students in Kahramanmaraş. From May 2017 to June 2017 a total of 960 students filled a “Rational Use of Drugs Questionnaire”.

**Results:**

The prevalence of practicing self-medication in students was 63.4%. The most common medicines that the students had consumed without prescription were analgesics by 39.5%, antibiotics by 36.9% and cold remedies by 24.0%. The rate of students who declared that they were familiar with RUD and “rational use of antibiotics” (RUA) was 45.9%. Reading/checking the instructions in the prospectus (OR = 1.529, 95% CI [1.176–1.990]), understanding the context of the prospectus (OR = 1.893, 95% CI [1.387–2.584]), compliance with the duration of antibiotic treatment (OR = 1.597, 95% CI [1.231–2.071]) and consulting a physician in case of a side effect (OR = 1.350, 95% CI [1.037–1.757]) were significantly higher among students who were familiar with RUD as compared to who were not.

**Discussion:**

Since the awareness of RUD among university students was found to be inadequate, it has critical importance to hold educational activities with the cooperation of physicians, health organizations, universities, non-governmental organizations and media to avoid negative consequences of irrational drug use and self-medication.

## Introduction

Self-medication refers to the use of medicines to treat self-diagnosed diseases without consulting any healthcare professionals. While responsible self-medication can be used to prevent and treat symptoms and ailments that do not need medical consultation or oversight, self-medication without sufficient knowledge may lead to serious obstacles in the rational use of drugs (RUD) (World Health Organization, 2000).

The first steps taken towards RUD were the description of essential drugs and national drug policy concepts by the World Health Assembly in 1975 (World Health Organization, 1975). Ten years after, during The Conference of Experts on Rational Use of Drugs in Nairobi, the current definition of rational use of medicines was concluded as “Rational use of medicines require that patients receive medications based on their clinical needs, in doses that meet their requirements, for an adequate period of time and at the lowest possible cost for patients and their community” (World Health Organization, 1987).

Irrational use of drugs is a serious problem throughout the world. Unnecessary drug use causes a heavy burden to the economy of developing countries such as Turkey (Karataş et al., 2012). In terms of the ratio of the gross national product allocated to health expenditure Turkey is last with 5.1% among OECD countries (OECD, 2015). However the share of total health expenditure allocated to drug expenditure in Turkey is over 20%, meaning drug costs hold a significant proportion of health expenditure (TURKSTAT, 2016).

Besides the economic burdens, there are also serious negative health effects of irrational drug use and self-medication. Previous research has demonstrated that usage of analgesics and antibiotics are the main subject of self-medication (Lukovic et al., 2014; Ibrahim et al., 2015; Zhu et al., 2016; Nayir et al., 2016). Improper use of analgesics is dangerous to health due to their toxic and harmful side effects (Ibrahim et al., 2015). The other most commonly observed mode of irrational drug use around the world is self-medication with antibiotics, which may lead to masking symptoms, treatment failure and development of drug resistance by bacteria (Zhu et al., 2016). Even though there are many reasons to promote RUD such as concerns about health and economy, still most of the responsibility belongs to physicians. However physicians’ efforts towards RUD alone are not sufficient; this should be supported by patient participation (Basaran & Akici, 2012).

Several studies investigating RUD and associated factors have shown that self-medication and irrational drug use habits are related with the level of education and people who have higher levels of education tend to indulge in self-medication more (Carrasco-Garrido et al., 2008; Foroutan & Foroutan, 2014; Garofalo, Di Giuseppe & Angelillo, 2015; Nayir et al., 2016). Therefore, the aim of this study is to assess the habits related to RUD and to estimate the prevalence of self-medication practices among university students in Kahramanmaraş, located at the Eastern Mediterranean Region of Turkey.

## Materials and Methods

### Study design and setting

This study is a cross-sectional study. The study took place in Kahramanmaraş, a city in the Eastern Mediterranean Region of Turkey. We aimed to evaluate self-medication prevalence and drug use habits of university students. In the study a “Rational Use of Drugs Questionnaire”, developed by the researchers and consisting of 24 questions was used. The first four questions pertain to the socio-demographic characteristics while the rest of the questions were related to health status, knowledge and manners of students with respect to RUD, and insensible consumption of procured over the counter (OTC) drugs. At least one positive response to one of the following three questions “Do you use others’ medicines or buy medicines from pharmacy without prescription?”, “Do you have medicines prescribed without being sick or buy and keep at home in case of need?” and “Do you use antibiotics on your own without a physician’s examination?” was considered to be practicing at least some form of self-medication (Nayir et al., 2016).

### Participants

The universe of this study was formed by 13,704 students who attended the non-healthcare faculties of Kahramanmaraş Sütçü İmam University Avşar Campus during the 2016–2017 academic years. Since there was no similar study carried exclusively on university students in this region, it was aimed to reach the maximum number of samples by assuming 50% prevalence of self-medication. Then, the sample size was calculated to be as 991 students in 95% confidence interval and with a 3% margin of error, using the Epi Info program. Before starting the study the questionnaires were pretested through a preliminary study and dysfunctional questions were corrected.

### Data collection and measurement

The calculated sample size was distributed by layered sampling method according to density of the faculties. Researchers carried out the distribution of questionnaires to the faculties and controlled whether the questionnaires were responded correctly. Data collection period was two months during May 2017 to June 2017. At the end of this period a total of 960 students (96.9% of targeted sample) have been reached out.

We used SPSS for Windows software for data analysis (SPSS, Chicago, IL, USA). Descriptive statistics were presented as frequencies and percentages in tables. Pearson chi-square test was applied to assess the results. The level of statistical significance was accepted as *p* < 0.05 and the estimated Odds Ratios (OR) were presented with 95% confidence interval. No data assignments were made to the missing data due to the unresponded questions. The data of non-missing values were presented.

### Ethical considerations

In this study, the data was used provided that the confidentiality of all participants is preserved. The study was approved by Scientific Researches Ethics Committee of Kahramanmaraş Sütçü İmam University (Decision date: 19.04.2017; Decision number: 02). Written informed consent was obtained from all participants and participation in the study was purely voluntary.

## Results

The number of students agreeing to participate in the study was 960. The rate of females was 55.6% (*n* = 534) and 89.6% of students were in the range of 18–23 years of age. 96.1% (*n* = 906) of students were single whereas 3.3% (*n* = 31) of them were married and 0.6% (*n* = 6) were divorcee or widow. 88.4% (*n* = 840) of the students had an economic perception of moderate level or better. The details about socio-demographic characteristics of students are presented in Table 1. 8.9% (*n* = 85) of the students had chronic diseases (Table 1). The most common chronic diseases included: chronic allergic diseases (asthma, sinusitis, rhinitis) 27.9% (24/85), migraine 10.5% (9/85) and ocular diseases (refractive errors, ocular hypertension) 9.4% (8/85). The rate of daily medicine users was 9.0% (*n* = 87). While 54.0% (47/87) of the daily medicine users were suffering from at least one chronic disease, it is striking that 46.0% (40/87) of them were using medicines on daily basis without even suffering from any chronic disease.

**Table 1:** Distribution of students according to socio-demographic characteristics.

Socio-demographic characteristics	*N*	%
**Gender**		
Female	534	55.6
Male	426	44.4
Total	960	100
**Age group (years)**		
18 to 20	308	32.5
21 to 23	542	57.1
24 to 26	72	7.6
Above 26	27	2.8
Total	949	100
**School**		
School of administrative and economic sciences	282	29.4
School of engineering and architecture	199	20.7
School of science and literature	196	20.4
School of theology	138	14.4
School of agriculture	67	7.0
School of education	45	4.7
School of forestry	33	3.4
Total	960	100
**Marital status**		
Single	906	96.1
Married	31	3.3
Divorcee or widow	6	0.6
Total	943	100
**Perceived economic status**		
Very bad	34	3.6
Bad	76	8.0
Moderate	602	63.5
Good	212	22.3
Very good	26	2.6
Total	950	100
**Existence of a chronic disease**		
Yes	85	8.9
No	865	91.1
Total	950	100

**Figure 1:**
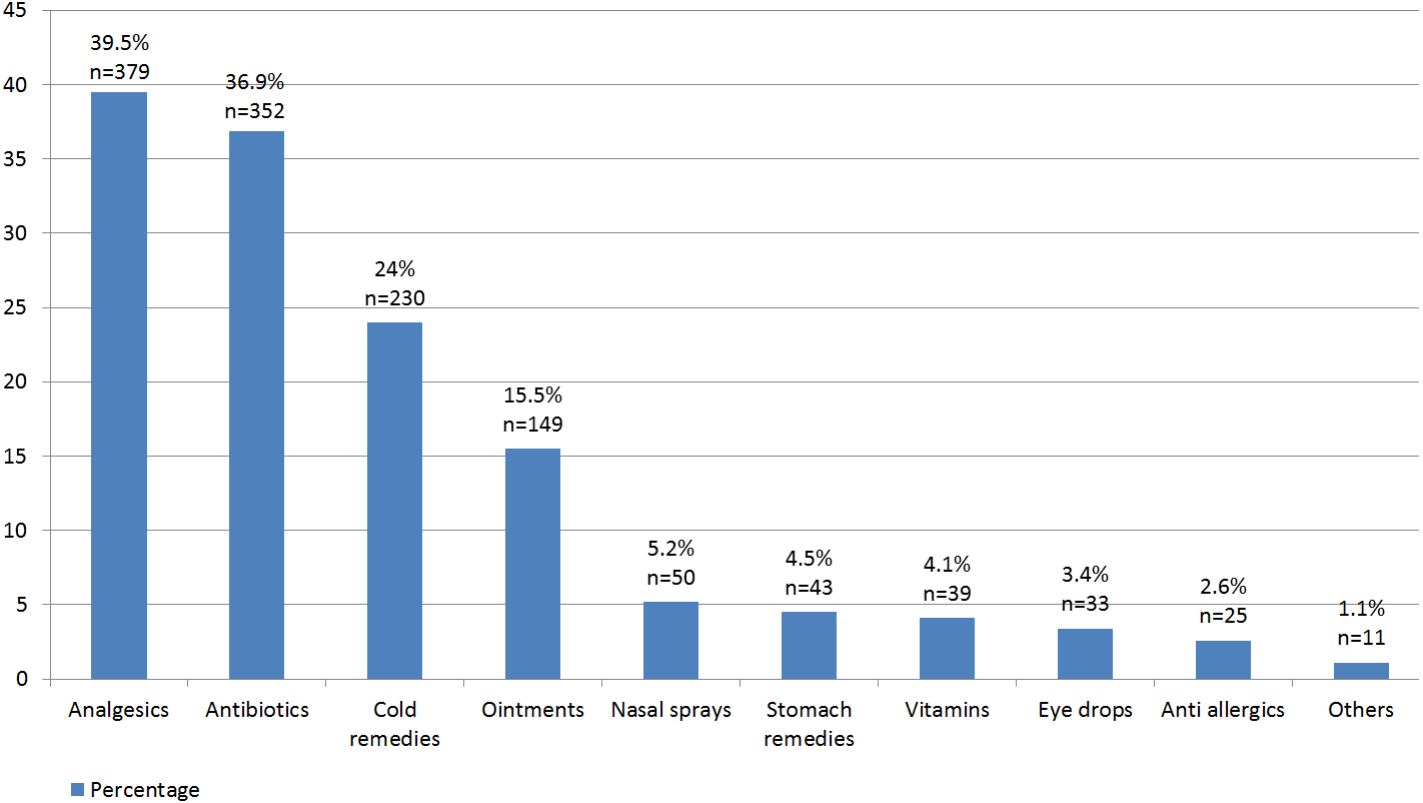
The most common medicines the students had consumed without prescription. The distribution of the types of medicines the students had consumed without prescription.

The rate of students who use others’ medicines or buy medicines from pharmacy without prescription was 43.8% (*n* = 420). The most common medicines that the students had consumed without prescription were analgesics by 39.5% (*n* = 379), antibiotics by 36.9% (*n* = 352) and cold remedies by 24.0% (*n* = 230) (Fig. 1). 73.1% of students had taken antibiotics in the past year however it is remarkable that more than half of the antibiotic consumption was without a physician’s examination. When the duration of the prescribed antibiotic usage of the students was examined, the rate of students who completed the antibiotherapy or quitted after consulting a physician/pharmacist was 43.7% (*n* = 411). 40.7% (*n* = 388) of the students had one to five boxes of unused or unfinished drugs at their residences and 39.0% (*n* = 371) of the students discarded one to three boxes of drugs within a year even without opening the box, as the expiry date had already lapsed. The majority of the students checked the instructions in the prospectus of the medications they used, however only 22.4% (*n* = 211) of them understood the information in the prospectus fully. 61.4% (*n* = 586) of the students stated that they would consult a physician in the case of a side effect. Finally, the prevalence of practicing some form of self-medication among students was revealed to be as 63.4% (*n* = 595). 45.9% (*n* = 437) of the students declared that they were familiar with RUD and “rational use of antibiotics” (RUA). The details of students’ drug use habits are presented in Table 2.

**Table 2:** Self-reported attitudes of students towards drug use.

Questions	*N*	%
**Do you use others’ medicines or buy medicines from pharmacy without prescription?**		
Yes	420	43.8
No	540	56.2
Total	960	100
**Do you have medicines prescribed without being sick or buy and keep at home in case of need?**		
Yes	191	20.3
No	750	79.7
Total	941	100
**Have you taken any antibiotics in the last 12 months?**		
Yes	690	73.1
No	254	26.9
Total	944	100
**Do you use antibiotics on your own without a physician’s examination?**		
Yes	352	36.9
No	602	63.1
Total	954	100
**How long do you use the antibiotics prescribed for you?**		
I quit after the symptoms disappear or a few days after I feel recovered	463	49.3
I quit after consulting a physician/pharmacist	114	12.1
I quit at the end of treatment	297	31.6
I quit a few days after whether I feel recovered or not	66	7.0
Total	940	100
**How many boxes of drugs do you have unused or unfinished in your house?**		
None	133	13.9
1–5	388	40.7
6–10	196	20.5
More than 10	238	24.9
Total	955	100
**Over a year, how many boxes of drugs are thrown away even without opening the box, since the expiry date has lapsed?**		
1–3	371	39.0
4–7	157	16.5
8–10	45	4.7
More than 10	69	7.3
None	309	32.5
Total	951	100
**Do you read/check the instructions in the prospectus of the medications you are using?**		
Yes, always	555	58.7
Yes, sometimes	329	34.9
No, I do not	60	6.4
Total	944	100
**How much do you understand about the information in the prospectus of the drug you are using?**		
I understand fully	211	22.4
I partially understand	692	73.3
I understand nothing	41	4.3
Total	944	100
**What do you do if you experience any side effects while taking medication?^a^**		
I quit medication	305	32.0
I quit the medicine and start a new one with the same effect	19	2.0
I consult to a pharmacist	46	4.8
I consult to a physician	586	61.4
I consult to my family	41	4.3
I do nothing	21	2.2
**Have you heard the expression of rational drug use and rational use of antibiotics before?**		
Yes	437	45.9
No	516	54.1
Total	953	100

**Notes.**

a Multiple responses, total does not add to 100%.

When the students who were familiar with the terms RUD and RUA were compared to those who were not, significant differences were observed in drug use habits. Reading/checking the instructions in the prospectus (OR = 1.529, 95% CI [1.176–1.990]), understanding context of the prospectus (OR = 1.893, 95% CI [1.387–2.584]), compliance to the duration of antibiotic treatment (OR = 1.597, 95% CI [1.231–2.071]) and consulting a physician in the case of a side effect (OR = 1.350, 95% CI [1.037–1.757]) were significantly higher among students who are familiar with the terms. However performing some form of self-medication did not differ between the participants who were aware of RUD and RUA and who were not. Additionally inappropriate drug use habits such as consulting family (OR = 0.418, 95% CI [0.207–0.845]) or doing nothing in case of a side effect (OR = 0.362, 95% CI [0.131–0.995]) were significantly less among students who are familiar with RUD and RUA. The comparison of students who were familiar with the terms RUD and RUA and those who were not according to their drug use habits are presented in Table 3.

**Table 3:** The comparison of students who were familiar with the terms “rational drug use” and “rational use of antibiotics” and those who were not according to drug use habits.

Drug use habits	Being familiar with rational drug use				*p*^a^	OR^b^
	Yes		No			
	*N*	%	*N*	%		
**Reading/checking the instructions in the prospectus**						
Always	277	64.4	277	54.2	**0.002**	**1.529 (1.176–1.990)**
Sometimes or never	153	35.6	234	45.8		
Total	430	100	511	100		
**Understanding context of the prospectus**						
Fully	122	28.3	88	17.3	**0.0001**	**1.893 (1.387–2.584)**
Partially or not at all	309	71.7	422	82.7		
Total	431	100	510	100		
**Performing some form of self-medication**						
Yes	274	64.0	320	63.0	0.745	1.045 (0.800–1.365)
No	154	36.0	188	37.2		
Total	428	100	508	100		
**Compliance with the duration of antibiotic treatment**						
Compliant	216	49.8	193	38.3	**0.0001**	**1.597 (1.231–2.071)**
Non-compliant	218	50.2	311	61.7		
Total	434	100	504	100		
**Actions taken in case of a possible side effect quitting medication**						
Yes	140	32.0	165	32.0	0.984	1.003 (0.763–1.318)
No	297	68.0	351	68.0		
Total	437	100	516	100		
**Quitting medication and starting a new one with the same effect**						
Yes	6	1.4	13	2.5	0.207	0.539 (0.203–1.429)
No	431	98.6	503	97.5		
Total	437	100	516	100		
**Consulting to a pharmacist**						
Yes	19	4.3	27	5.2	0.525	0.823 (0.451–1.502)
No	418	95.7	489	94.8		
Total	437	100	516	100		
**Consulting to a physician**						
Yes	285	65.2	300	58.1	**0.025**	**1.350 (1.037–1.757)**
No	152	34.8	216	41.9		
Total	437	100	516	100		
**Consulting to family**						
Yes	11	2.5	30	5.8	**0.012**	**0.418 (0.207–0.845)**
No	426	97.5	486	94.2		
Total	437	100	516	100		
**Taking no action**						
Yes	5	1.1	16	3.1	**0.040**	**0.362 (0.131–0.995)**
No	432	98.9	500	96.9		
Total	437	100	516	100		

**Notes.**

a Pearson Chi-Square Test; *α*:0,05.

b All ORs were obtained through bivariate analyses.

Bolded values are those which have statistical difference between groups at *p* < 0.05 level.

## Discussion

The aim of our study has been to assess the habits related to RUD and to estimate the prevalence of self-medication practices among students in a university in Turkey. It is a well-known fact that self-medication is widespread worldwide. In previous studies, the reported prevalence of self-medication varied according to the targeted population. Several studies conducted on the general public reported this rate between 50–60% (Foroutan & Foroutan, 2014; Papakosta, Zavras & Niakas, 2014; Azami-Aghdash et al., 2015; Nayir et al., 2016). However in studies focusing on university students, the rates were rising, particularly among those who study medicine and other health related fields (Sawalha, 2008; Klemenc-Ketis, Hladnik & Kersnik, 2010; Sharma et al., 2015). In the present study, which is carried out on non-healthcare students, the estimated prevalence of practicing some form of self-medication in students was 63.4%. In accordance to our findings, a recent meta-analysis has reported that the prevalence of self-medication among students as 67% which was higher than its mean public rate (53%) (Azami-Aghdash et al., 2015).

It was found that 8.9% of the students had chronic diseases and the rate of daily medicine users was 9.0%. It is noteworthy that 46.0% of the daily medicine users were using medicines without even suffering from any chronic disease. In a study carried out on general public in Elazığ, Turkey, the rate of using medicines on daily basis without having any chronic disease 26.8% (Nayir et al., 2016), yet another finding which indicates self-medication is more frequent among university students than the general public.

The most common medicines that the students had consumed without prescription were analgesics by 39.5%, antibiotics by 36.9% and cold remedies by 24.0%. Although, the rates and rankings differ, studies carried out in Spain, Palestine, Finland, Nigeria, Iran and Turkey revealed that the most common medicines that have been subject to self-medication were analgesics, antibiotics and cold remedies (Hayran, Karavus & Aksayan, 2000; Turunen et al., 2005; Carrasco-Garrido et al., 2008; Sawalha, 2008; Auta et al., 2012; Ahmadi et al., 2016). This concludes that self-medication presents a similar pattern irrespective of any country in the globe.

Haphazard antibiotic use is associated with bacterial resistance development which is a danger more important than its economic burden. In this study, it is found that more than one in third of antibiotic consumption was without a physician’s examination. In a study carried out in İzmir, Turkey in 2005, the prevalence of self-medication with antibiotics was reported as 44.9% among non-healthcare university students (Buke et al., 2005). This rate is higher than in the present study. It is thought that the decrease in this rate was due to the restrictions applied on antibiotics sales in pharmacies recently. Nevertheless, it may be concluded that still uncontrolled use of antibiotics is frequent despite the regulations. In addition the rate of students discontinuing antibiotherapy was 56.3%. Our findings are in accordance with the previous studies that reported that change of dosage or antibiotics and discontinuation after disappearance of symptoms during self-medication with antibiotics were common among university students (El Ezz & Ez-Elarab, 2011; Zhu et al., 2016).

It is found that 40.7% of the students had one to five boxes of unused or unfinished drugs at their residences and 39.0% of the students discarded one to three boxes of drugs within a year even without opening the box, as the expiry date had already lapsed. This finding is a typical example of economic damage caused by irrational drug use. In Turkey, an average 7% of the medicines in pharmacies are disposed of due to expiry date. The expiry dates of 60% of the drugs that were kept at home come even without opening the box. The cost of the drug thrown into garbage is approximately 500 million dollars annually, which brings a heavy burden to the economy of a developing country such as Turkey (Pınar, 2012)

In this study the majority of the students reported reading the instructions in the prospectus of the medications they used. This is in accordance with a study conducted in Ireland, which reported over 80% of the participants read the instructions provided with the medicine (Wazaify et al., 2005). However, only 22.4% of the students in our study remarked to understand the information in the prospectus fully. Consistently, in a study conducted in İstanbul, Turkey understanding the drug leaflet was found to be 30.6% in non-health related university students (Akici & Basaran, 2013). Similarly, in a study carried out on university students in Thailand, understanding the context of the medicines’ prospectus was found inadequate (Burapadaja, Jamroendararasame & Sanguansermsri, 2002). If understanding the information in the prospectus is insufficient even in university students, for those who have low education level, understanding level should be much lower. It is suggested that the content of medicines’ prospectus should be simple, easy and understandable.

It is found that, in the case of a side effect, 61.4% of the students stated that they would consult to a physician; however only 4.8% of the students stated that they would consult to a pharmacist. In another study conducted on general public in Ankara, Turkey, it was reported that 10.1% of respondents remarked consulting to a pharmacist if they encounter a side effect (Özçelikay, 2001), which is a higher rate than we observed in our study. A study from Estonia reported that trust towards a pharmacist as a drug information source was lower among younger people (Villako, Volmer & Raal, 2012). Our study population is composed of university students, which may explain why the rate of consulting to a pharmacist in case of a side effect is lower.

In the present study, 45.9% of the students declared that they were familiar with the terms RUD and RUA. This rate is very similar to that obtained in a previous study from Elazığ, Turkey conducted on the general public (Nayir et al., 2016). These findings indicate that awareness of RUD is inadequate in Turkey. Although hearing the terms RUD and RUA were not at plausible levels in this study, it is noteworthy that appropriate drug use habits such as reading or checking the instructions in the prospectus, understanding context of the prospectus, compliance to the duration of antibiotic treatment and consulting to a physician in case of a side effect were significantly higher among students who are familiar with the terms. On the contrary inappropriate drug use habits such as consulting to family or doing nothing in case of a side effect were significantly higher among students who are not familiar with the terms RUD and RUA. There is evidence in the literature that positive attitudes regarding self-medication can be developed through education (Teramachi, 2013).

To the best of our knowledge, this study by far has the largest sample size in university settings as compared to previous studies carried out in Turkey. Another strength of the present study is that it provides useful data on self-medication with all types of medicines, as the previous studies from Turkey regarding self-medication, mainly focused on antimicrobial agents. Lastly, with the exclusion of medicine and other health related faculties, a certain prevalence of self-medication among non-healthcare students could be estimated. However several limitations may be addressed for our study. One of the limitations of the present study is that, due to time and resource limitations, the study was carried out only on the selected sample. Another limitation is that, as it is a survey study, memory factors may affect the responses to the questionnaires. Also, the data collection period was two months which may obstruct assessing the temporal characteristics of drug usage.

## Conclusion

The research on the concept of RUD has been increasing since 1970’s. In Turkey, however, researchers have begun to focus on this issue in the last two decades. Even so, significant steps have been taken towards RUD. Recent application of restrictions to antibiotics sales by the government in pharmacies was one of these steps. Since then, there has been a decrease in self-medication with antibiotics as shown in the present study. The awareness of RUD among university students was found to be inadequate. As demonstrated in this study, appropriate drug use habits were more common in those familiar with the terms RUD and RUA. As a result it has critical importance to hold educational activities with the cooperation of physicians, health organizations, universities, non-governmental organizations and media in order to avoid negative consequences of irrational drug use and self-medication. We believe this study may also provide epidemiological data for further studies regarding RUD.

##  Supplemental Information

10.7717/peerj.3990/supp-1 Supplemental Information 1 Raw data

10.7717/peerj.3990/supp-2 Supplemental Information 2 Questionnaire

## Additional Information and Declarations

### Data Availability

The following information was supplied regarding data availability: The raw data was uploaded as a Supplemental File.
