# Supplementary material for: Self-medication practices and rational drug use habits among university students: a cross-sectional study from Kahramanmaraş, Turkey
Source: PeerJ. 2017 Nov 1;5:e3990. doi: 10.7717/peerj.3990 (PMC5671114; doi:10.7717/peerj.3990)
Supplement: Supplemental Information 2 [file peerj-05-3990-s002.docx]

**KAHRAMANMARAŞ SÜTÇÜ İMAM UNIVERSITY FACULTY OF MEDICINE**

**Rational Drug Use Questionnaire**

1. How old are you?............ Your faculty? ………………................................................... Your class?…..
2. Your gender?

Female Male

1. What is your marital status?

Single Married Widowed/divorcee

4. How do you perceive your economic status?

Very bad Bad Moderate Good Very good

1. Do you have any chronicle disease?

Yes (Write the name of disease :………………………………) No

1. Do you have any medicine you use on daily basis?

Yes (Write the name of the medicine :…………………..…) No

1. Do you use others’ (e.g. your friends’, siblings’, neighbors’ medicine)medicines which were not presribed for you or buy medicines from pharmacy without a prescription? **If your answer is no, please skip to 10^th^ question.**

Yes No

1. **If your answer is yes to 7^th^ question,** which medicines do you use?

Pain killers

Antibiotics

Cold remedies

Vitamins

Stomach remedies

Eye drops

Nasal sprays

Allergy medicines

Ointments

All of the above

Other (…………………………………………………………………….)

1. **If your answer is yes to 7th question,** what is the reason of using medicines on your own?

It is cheaper It is easier to bu medicine than applying to a physician

I do not trust the physician Other (……………………………..)

1. What do you do to the family members’ medicines which are left unfinished after treatment?

I store them to use when necessary

I give them to health institutions

I give them to pharmacy

I give them to acquaintances who want them

I discard them

Other

1. About how many boxes of unused or unfinished medicines are there at your house?

None

1-5

6-10

Over 10

1. About how many boxes of medicines are discarded each year without opening the box due to the expiry date lapsed at your house?

1-3

4-7

8-10

Over 10

None discarded.

1. Do you have medicines prescribed without being sick or buy and keep at home in case of need?

Yes No

1. Have you taken any antibiotics in the last 12 months?

Yes No

1. Do you use antibiotics on your own without a physician's examination?

Yes; I do

Yes; I do but I quit using when I feel good.

No; I do not use without a physician's examination

1. In which occasions do you use antibiotics?

In the presence of nasal discharge When I feel tired and weak

When I start coughing For not infecting anyone

In the presence of toothache When I am diarrhea

When there is burning and itching in my eyes In the presence of postnasal drip

When I have nausea or vomiting When my skin is wounded

When I have fever Other ( Please specify …………………………………)

1. How do you choose the antibiotic you are using?

By suggestion of pharmacist

By suggestions of family members

By suggestions of friends

My personal experience

Previous prescription

By information obtained from the internet or television

1. How long do you use the antibiotics prescribed for you?

I quit a few days after whether I feel recovered or not I quit after the symptoms disappear

A few days after I feel recovered I quit after consulting a physician/pharmacist

I quit at the end of treatment After finishing the antibiotics Other(……………………)

1. Do you read/check the instructions in the prospectus of the medications you are using?

Yes, always Yes, sometimes

No, I do not

1. How much do you understand about the information in the prospectus of the drug you are using?

I understand fully I partially understand I understand nothing

1. Have you experienced any side effects while taking medication?

Yes (If yes, please specify…………………………………………………) No

1. What do you do if you experience any side effects while taking medication?

I quit medication I quit the medicine and start a new one with the same effect

I consult to a pharmacist I consult to a physician

I consult to my family Other (…………………….)

I take no action

1. Have you heard the expression of rational drug use and rational use of antibiotics before?

Yes No

1. **(If yes)** Please mark where you heard the expressions “rational drug use” and “rational use of antibiotics”. (You can mark more than one choice)

Pharmacy Television Social media

Hospital Internet Newspapers

Other (Please specify……………………………) ***Thank you for participating.***
